# Supplementary figures and images for: Unveiling the signaling network of FLT3-ITD AML improves drug sensitivity prediction
Source: eLife. 2024 Apr 2;12:RP90532. doi: 10.7554/eLife.90532 (PMC10987088; doi:10.7554/eLife.90532)

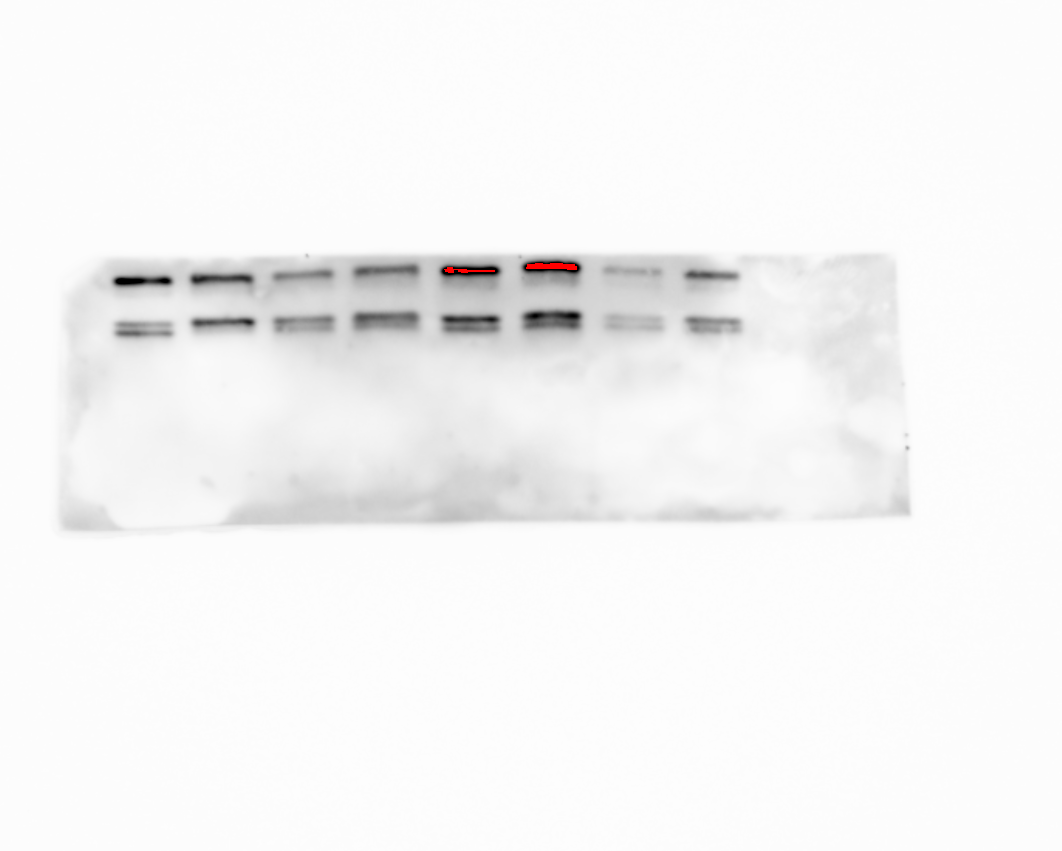

Supplement: Figure 4—source data 1. [file elife-90532-fig4-data1.zip › blots figure 4 raw/cdk2(Chemiluminescence).tif]

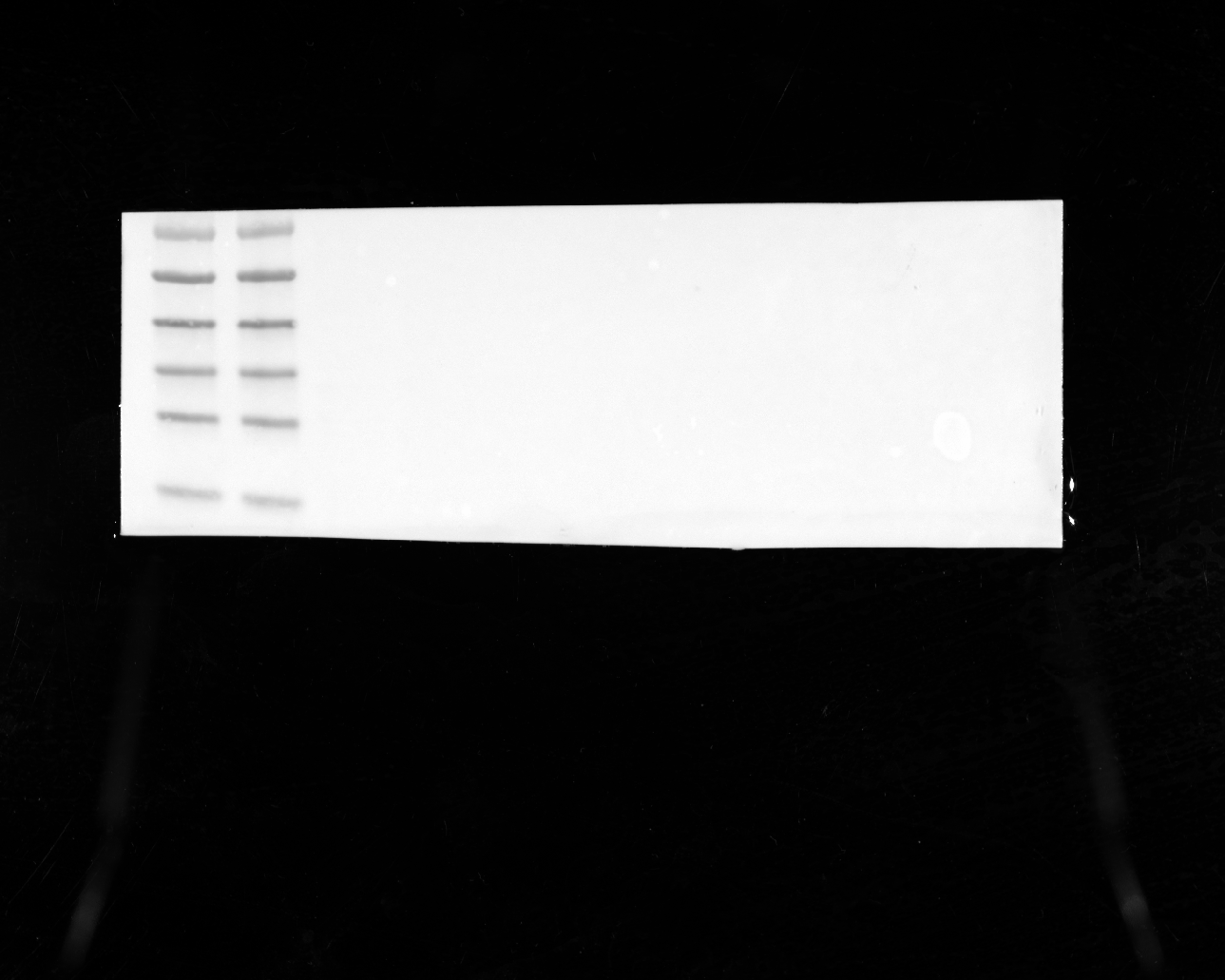

Supplement: Figure 4—source data 1. [file elife-90532-fig4-data1.zip › blots figure 4 raw/jnk(Colorimetric).tif]

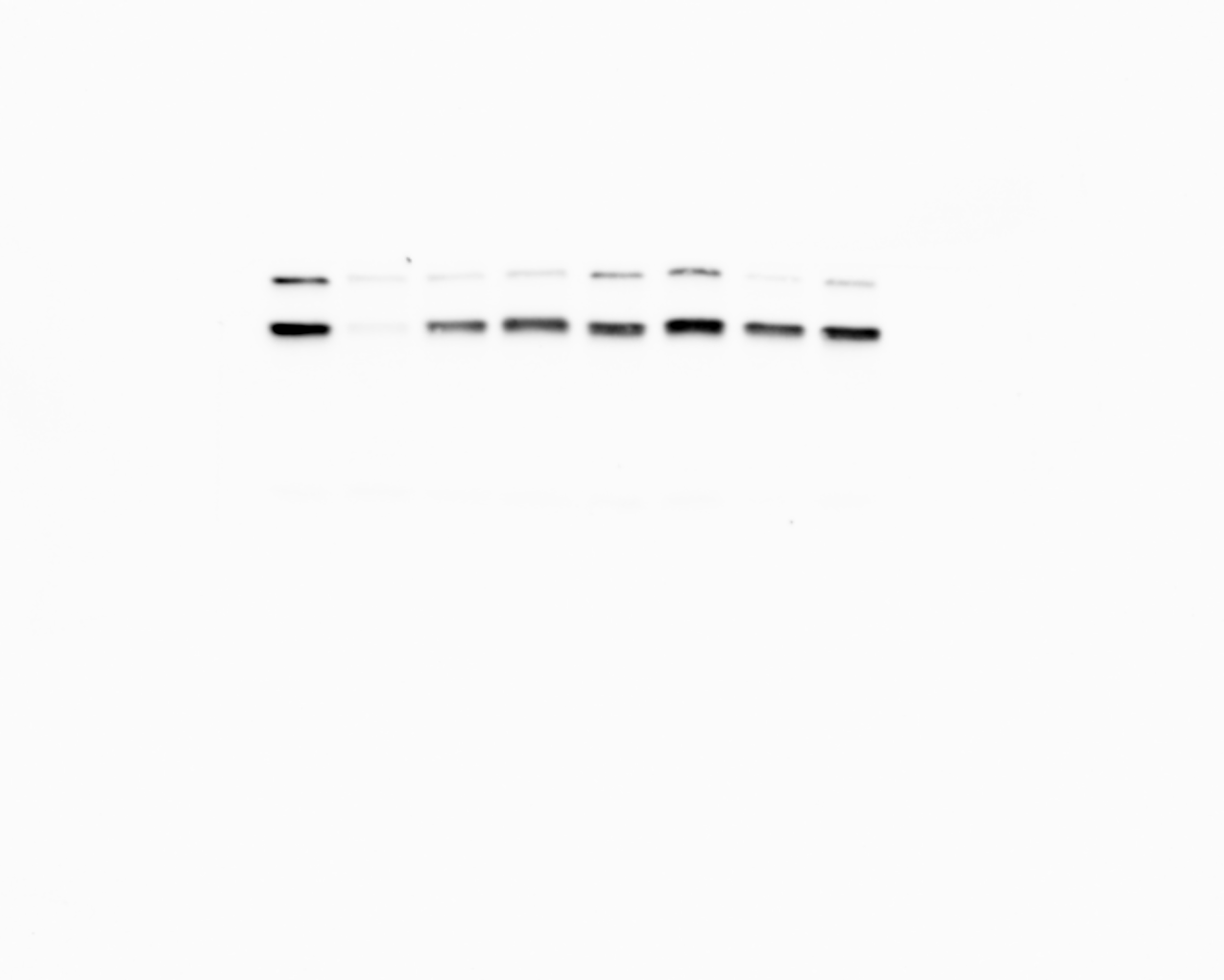

Supplement: Figure 4—source data 1. [file elife-90532-fig4-data1.zip › blots figure 4 raw/p cdk2 (Chemiluminescence).tif]

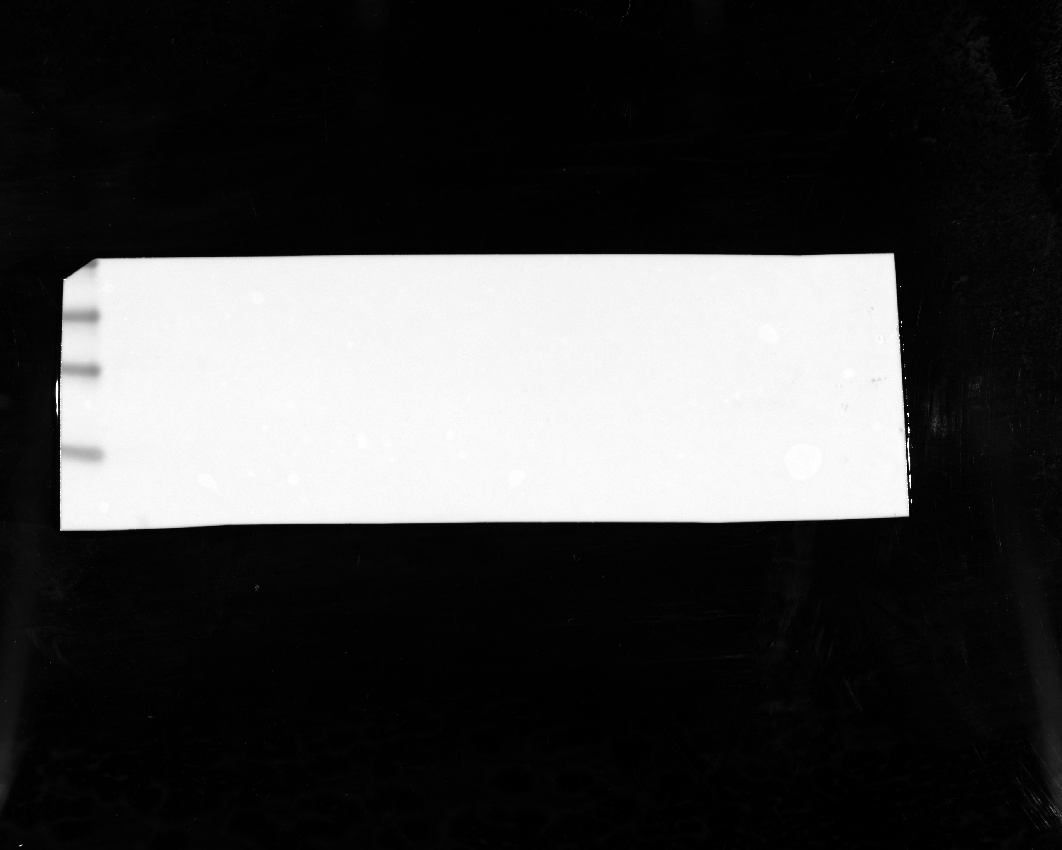

Supplement: Figure 4—source data 1. [file elife-90532-fig4-data1.zip › blots figure 4 raw/cdk2 (Colorimetric).tif]

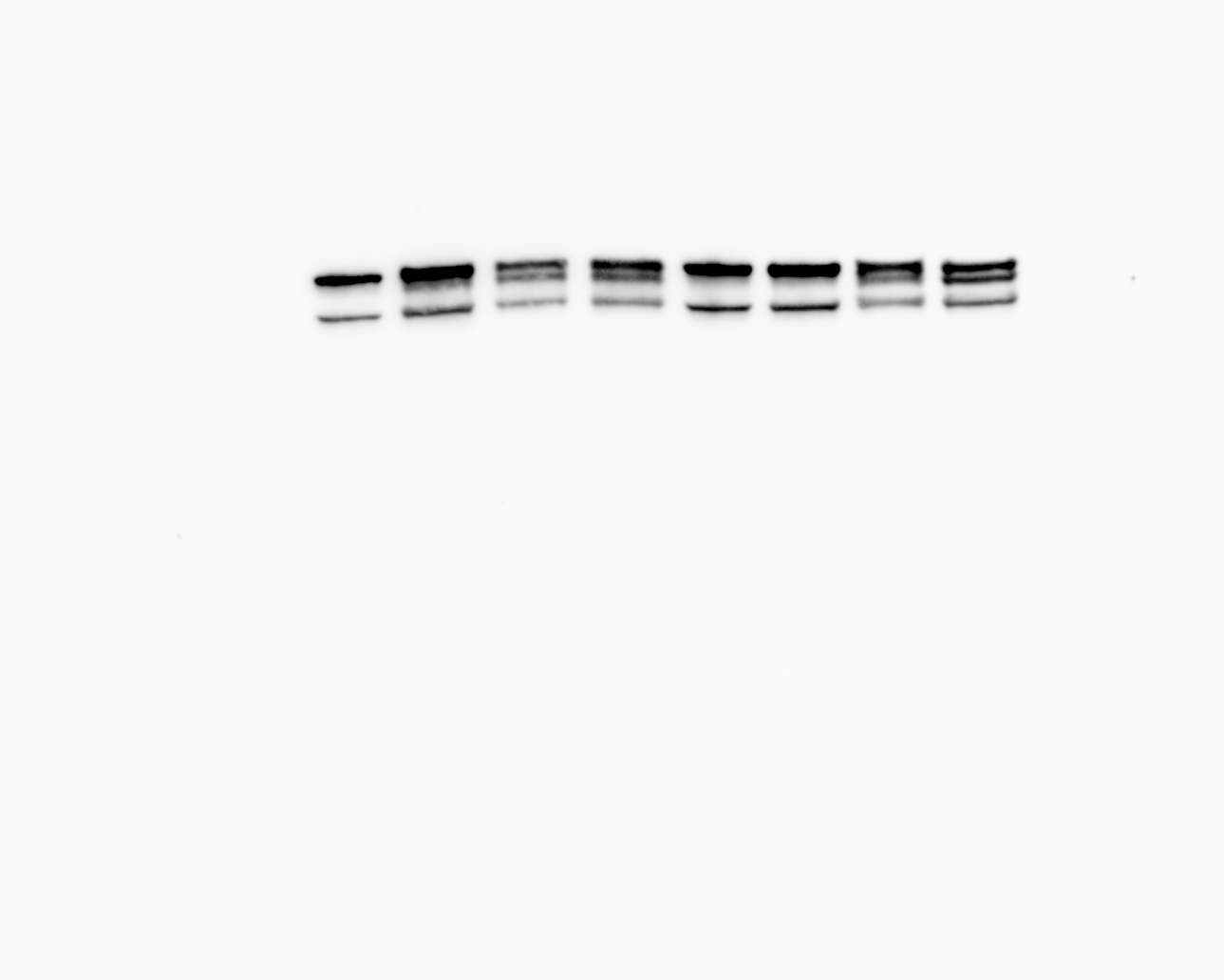

Supplement: Figure 4—source data 1. [file elife-90532-fig4-data1.zip › blots figure 4 raw/jnk(Chemiluminescence).tif]

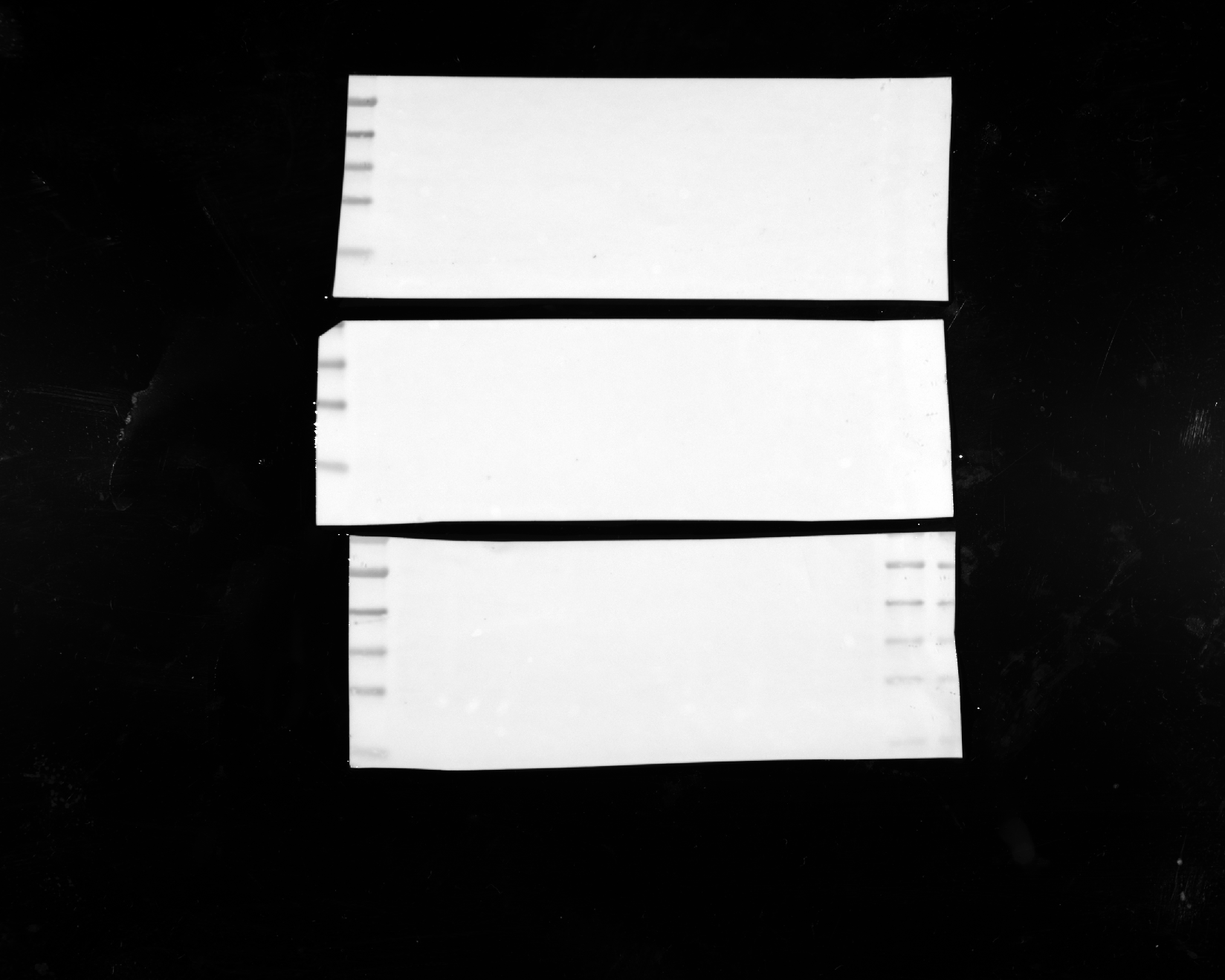

Supplement: Figure 4—source data 1. [file elife-90532-fig4-data1.zip › blots figure 4 raw/pcdk1 T161(Colorimetric).tif]

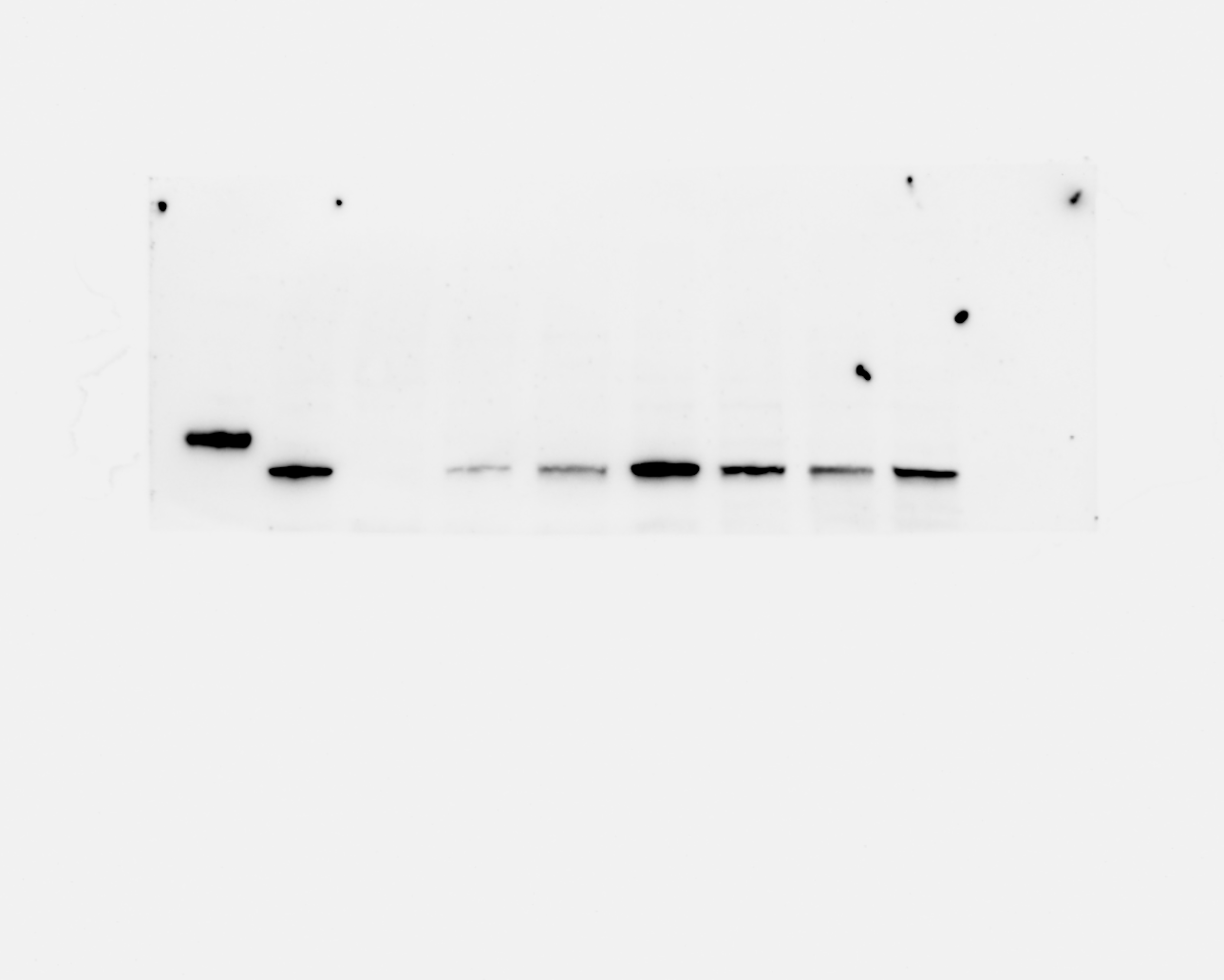

Supplement: Figure 4—source data 1. [file elife-90532-fig4-data1.zip › blots figure 4 raw/cyclin b1(Chemiluminescence).tif]

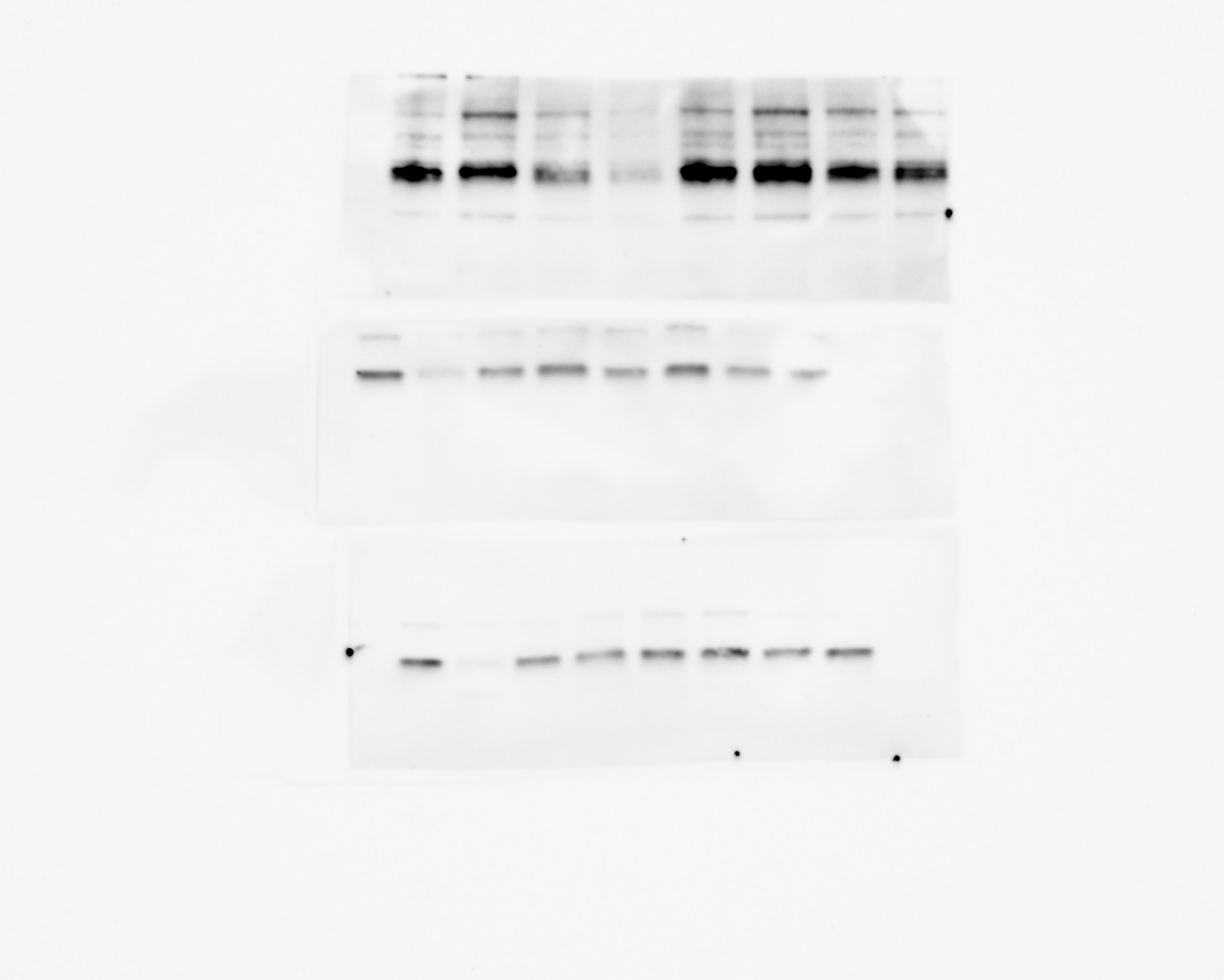

Supplement: Figure 4—source data 1. [file elife-90532-fig4-data1.zip › blots figure 4 raw/pcdk1 T161 (Chemiluminescence).tif]

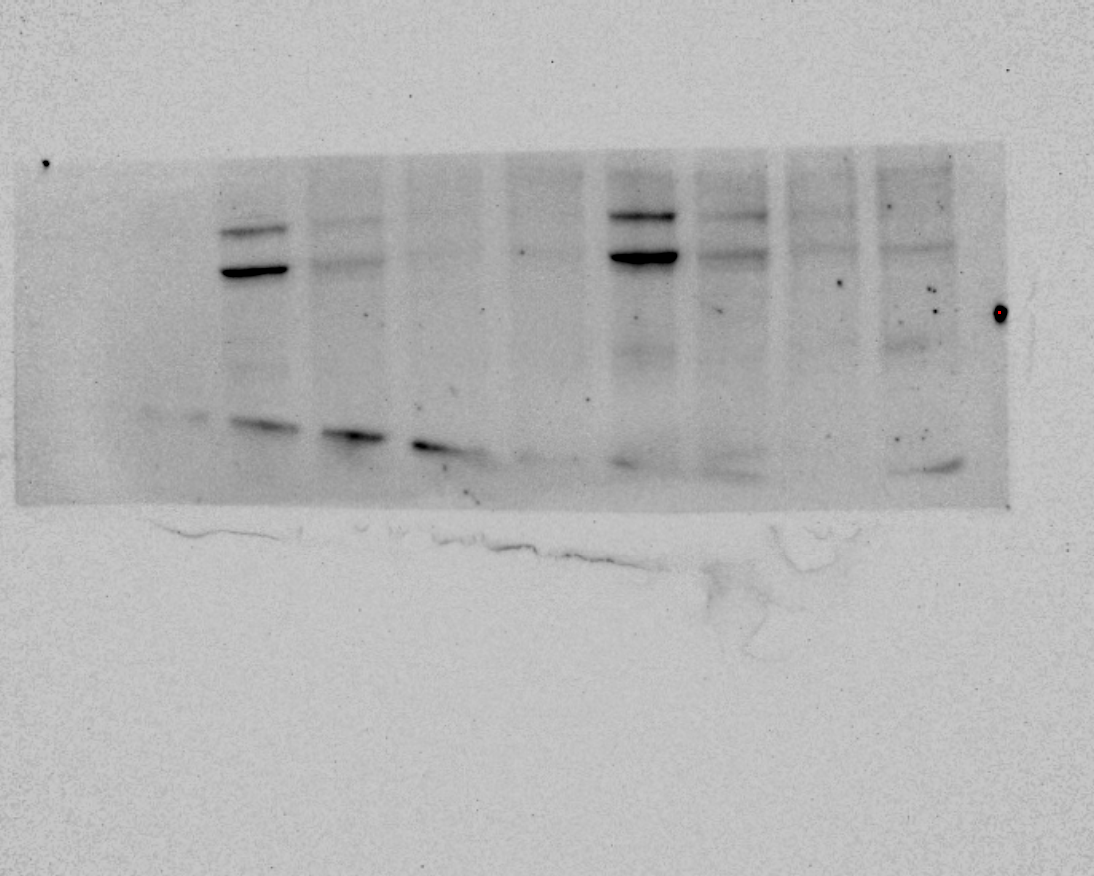

Supplement: Figure 4—source data 1. [file elife-90532-fig4-data1.zip › blots figure 4 raw/p jnk(Chemiluminescence).tif]

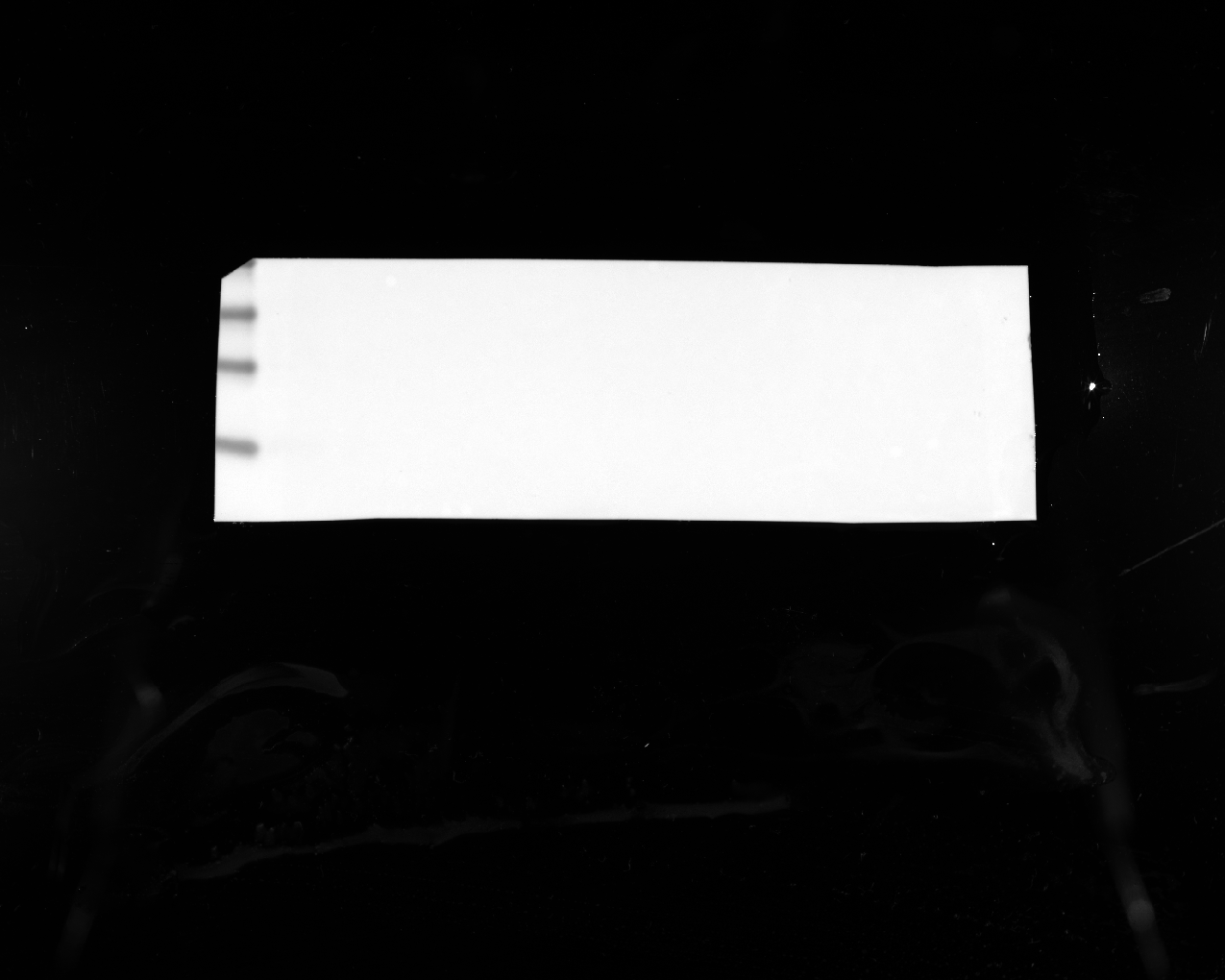

Supplement: Figure 4—source data 1. [file elife-90532-fig4-data1.zip › blots figure 4 raw/p cdk2 (Colorimetric).tif]

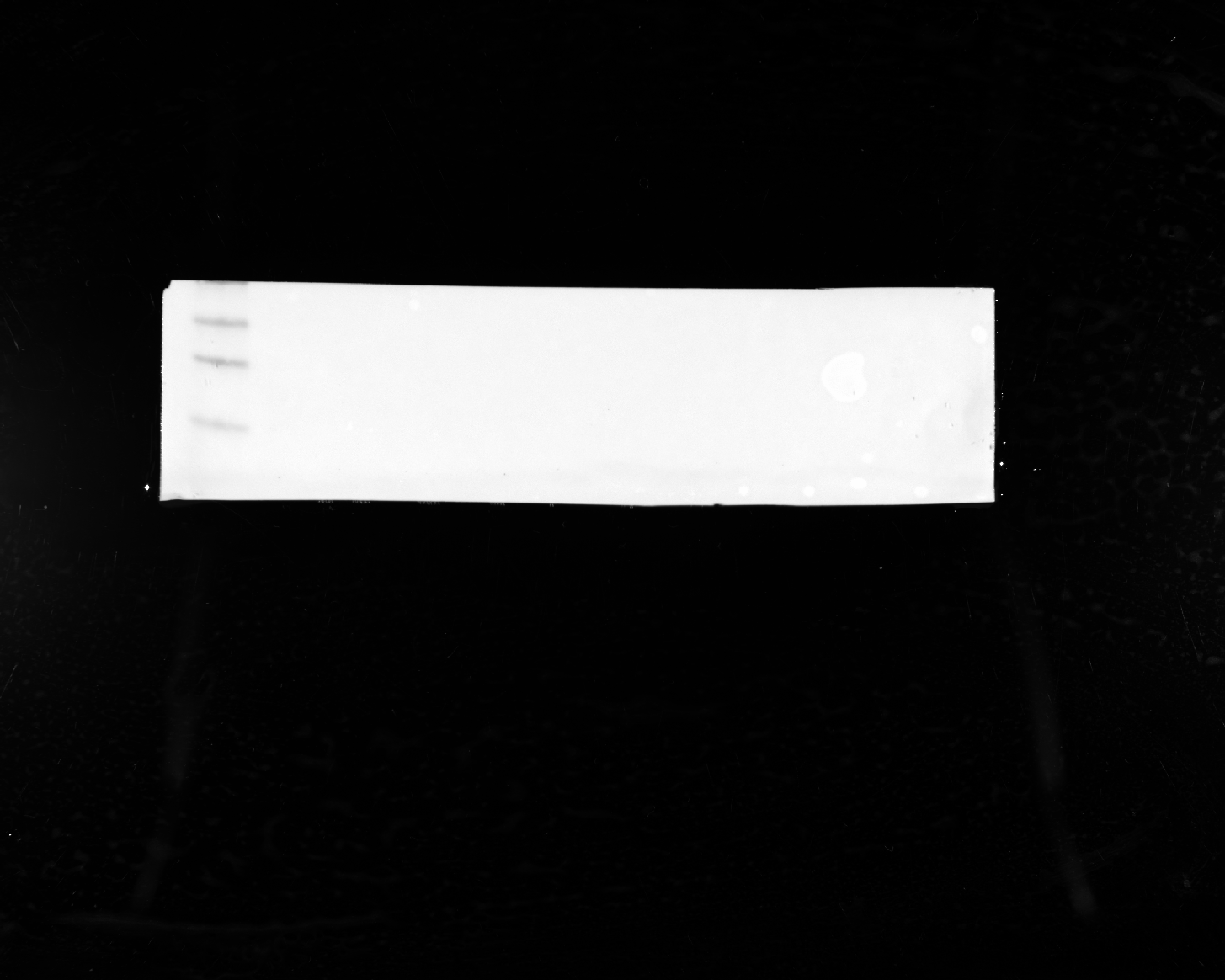

Supplement: Figure 4—source data 1. [file elife-90532-fig4-data1.zip › blots figure 4 raw/cdk1 (Colorimetric).tif]

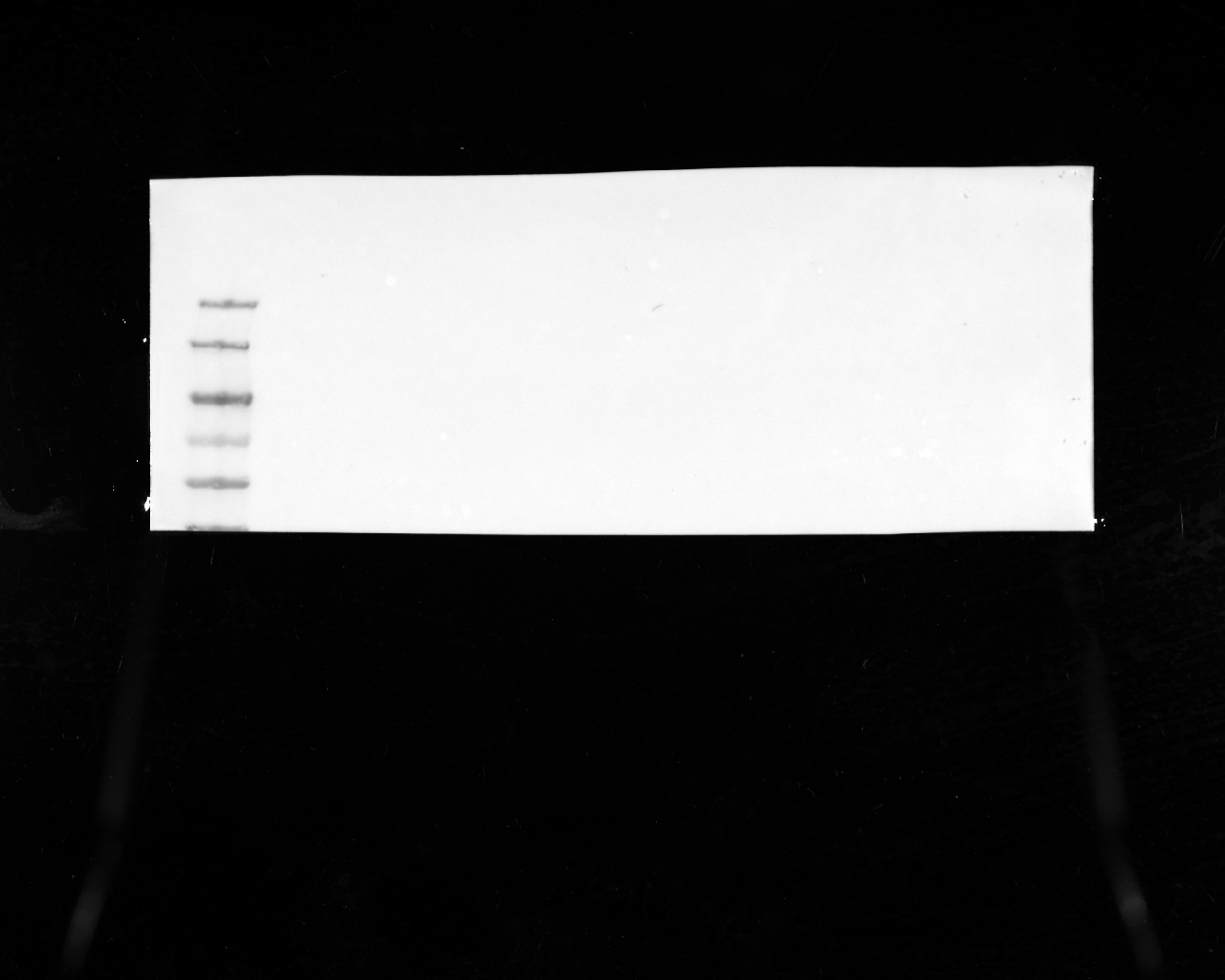

Supplement: Figure 4—source data 1. [file elife-90532-fig4-data1.zip › blots figure 4 raw/cyclin b1(Colorimetric).tif]

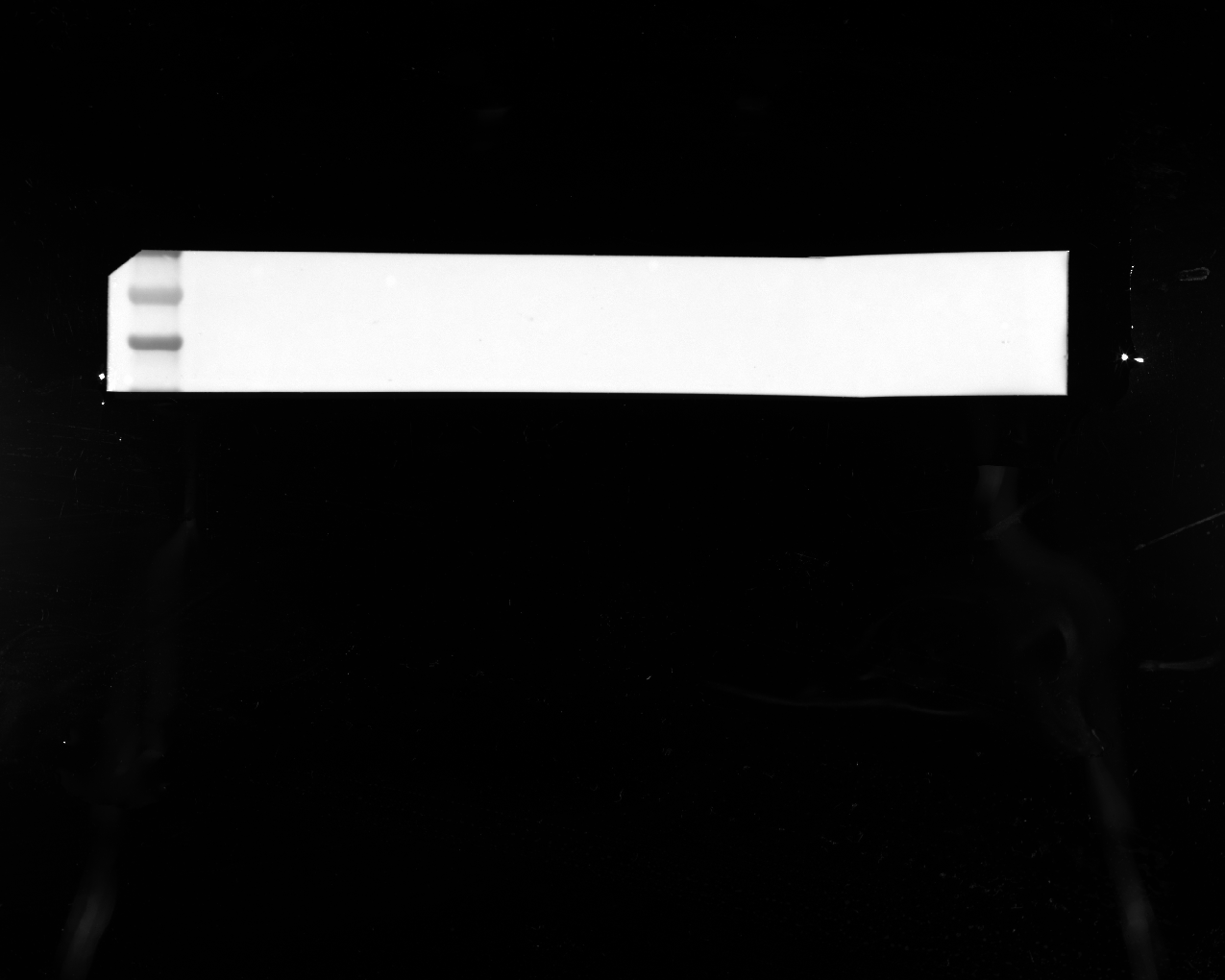

Supplement: Figure 4—source data 1. [file elife-90532-fig4-data1.zip › blots figure 4 raw/cyclinE2 (Colorimetric).tif]

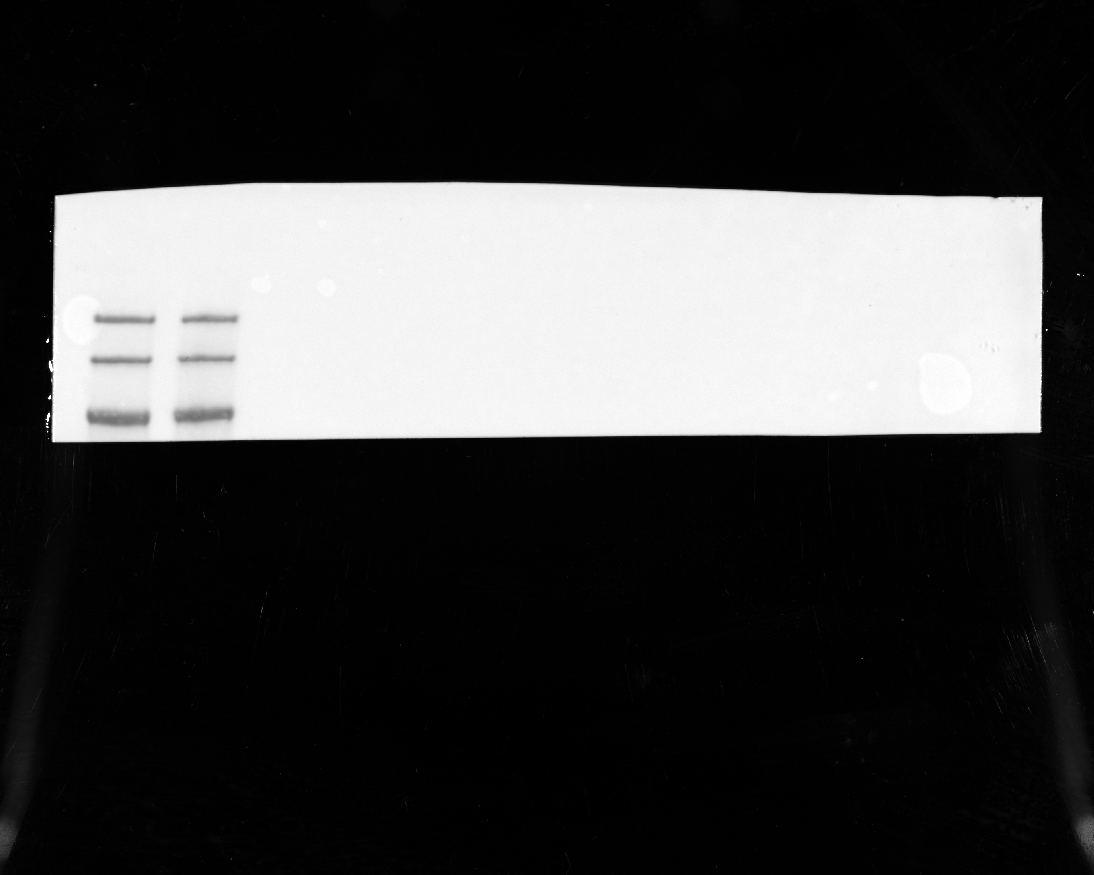

Supplement: Figure 4—source data 1. [file elife-90532-fig4-data1.zip › blots figure 4 raw/vinculin (Colorimetric).tif]

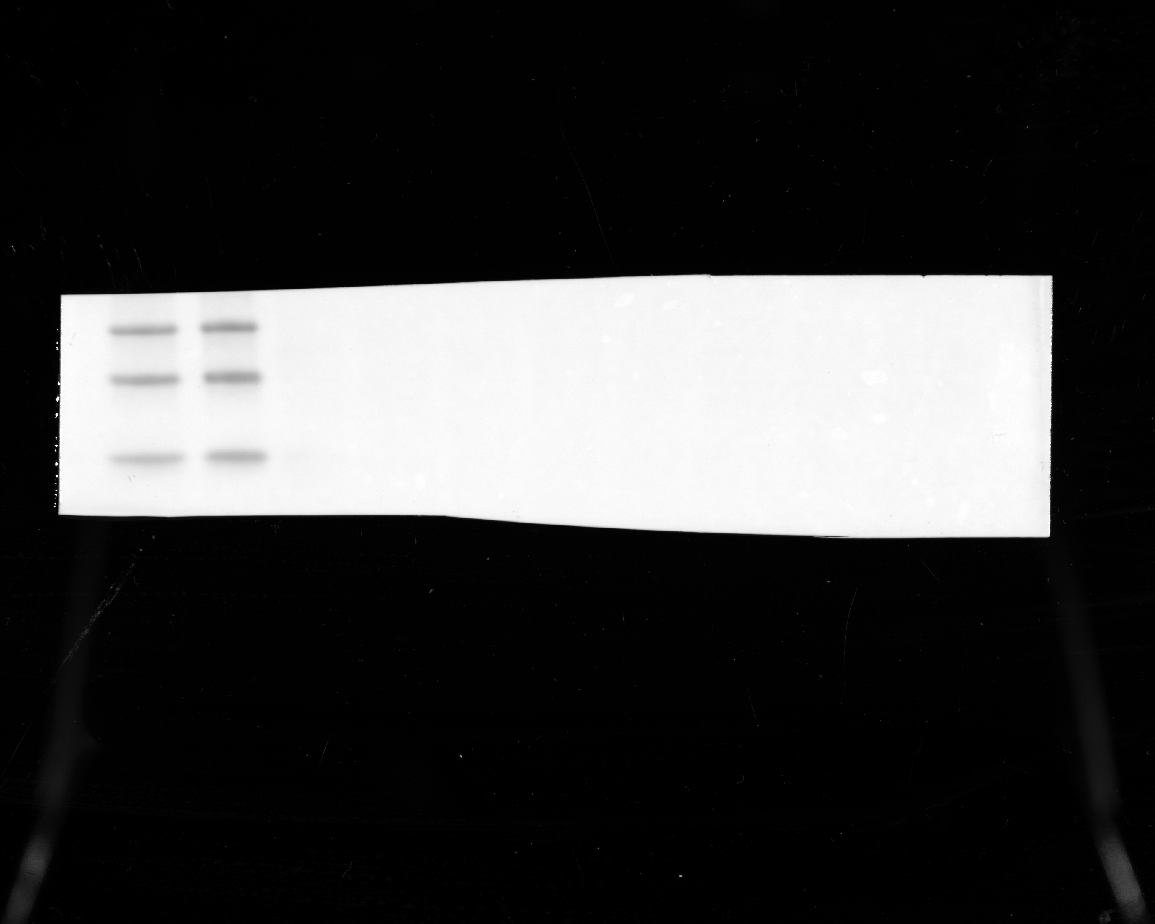

Supplement: Figure 4—source data 1. [file elife-90532-fig4-data1.zip › blots figure 4 raw/pCDK1 tyr15 (Colorimetric).tif]

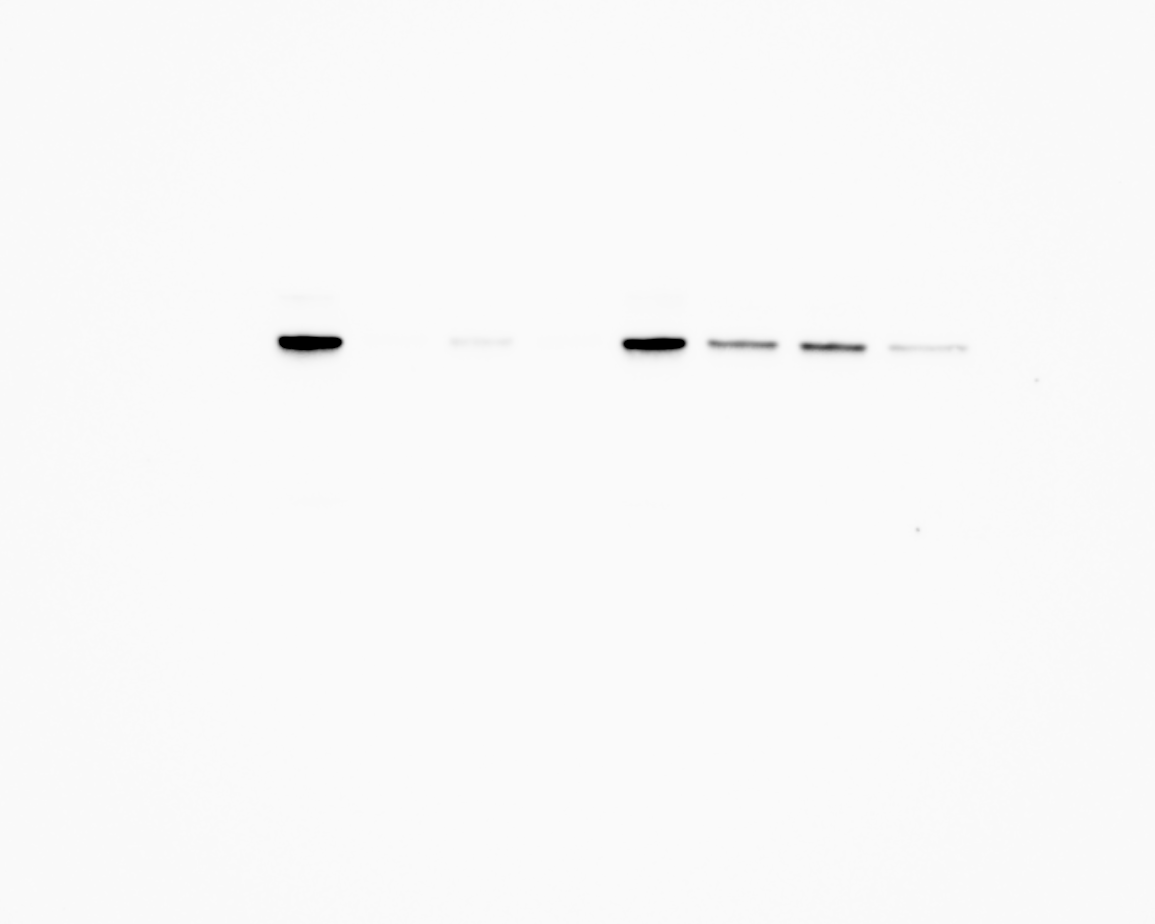

Supplement: Figure 4—source data 1. [file elife-90532-fig4-data1.zip › blots figure 4 raw/pCDK1 tyr15(Chemiluminescence).tif]

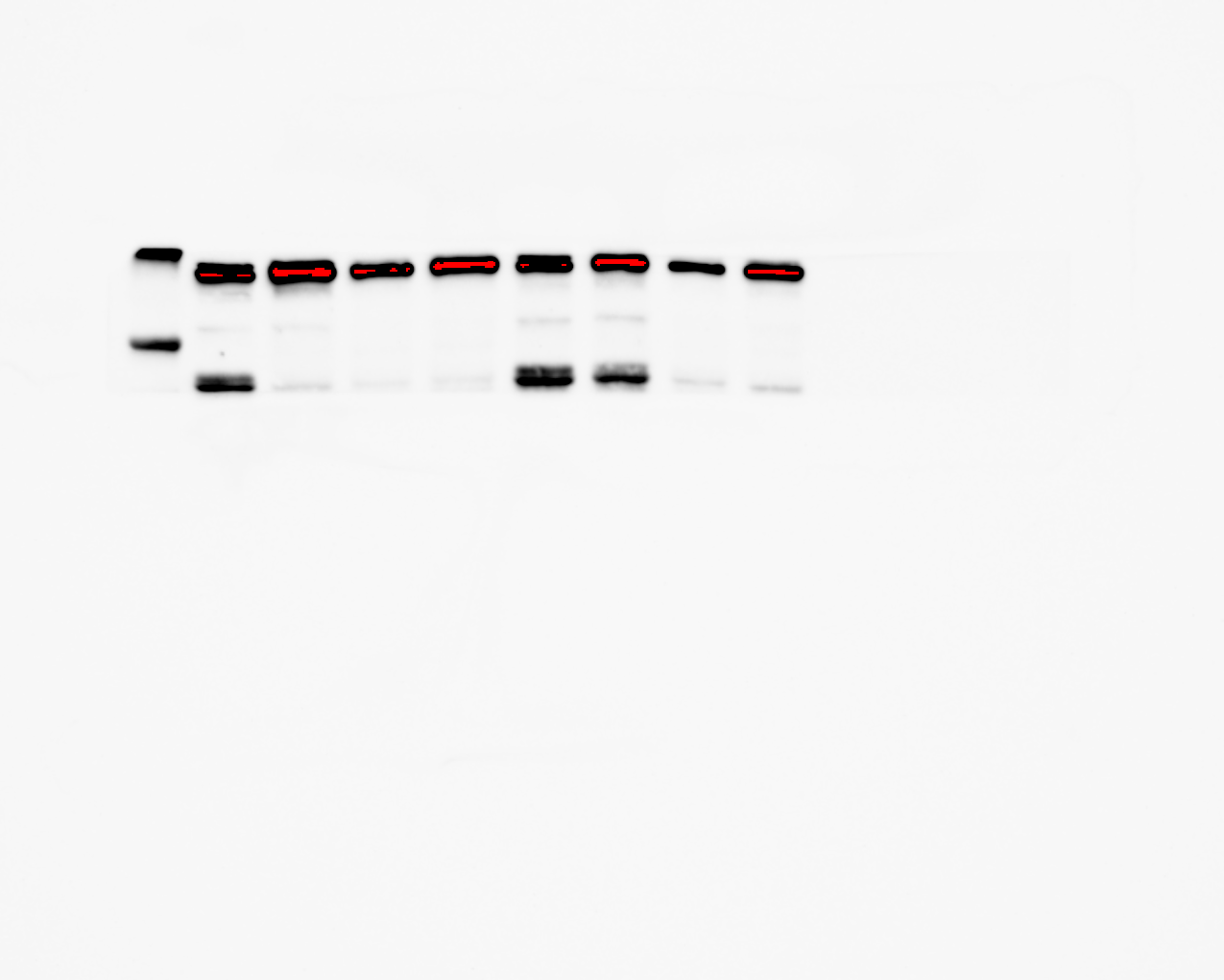

Supplement: Figure 4—source data 1. [file elife-90532-fig4-data1.zip › blots figure 4 raw/cyclinE2 (Chemiluminescence).tif]

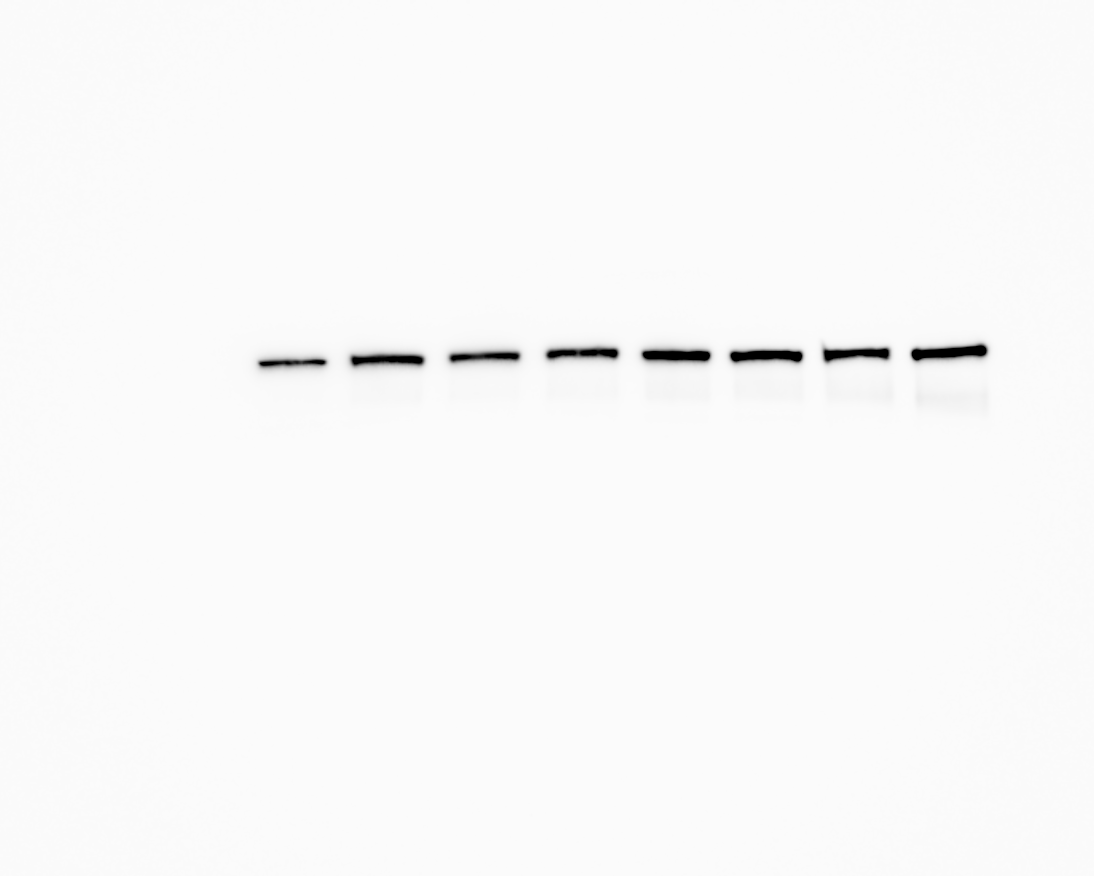

Supplement: Figure 4—source data 1. [file elife-90532-fig4-data1.zip › blots figure 4 raw/vinculin (Chemiluminescence).tif]

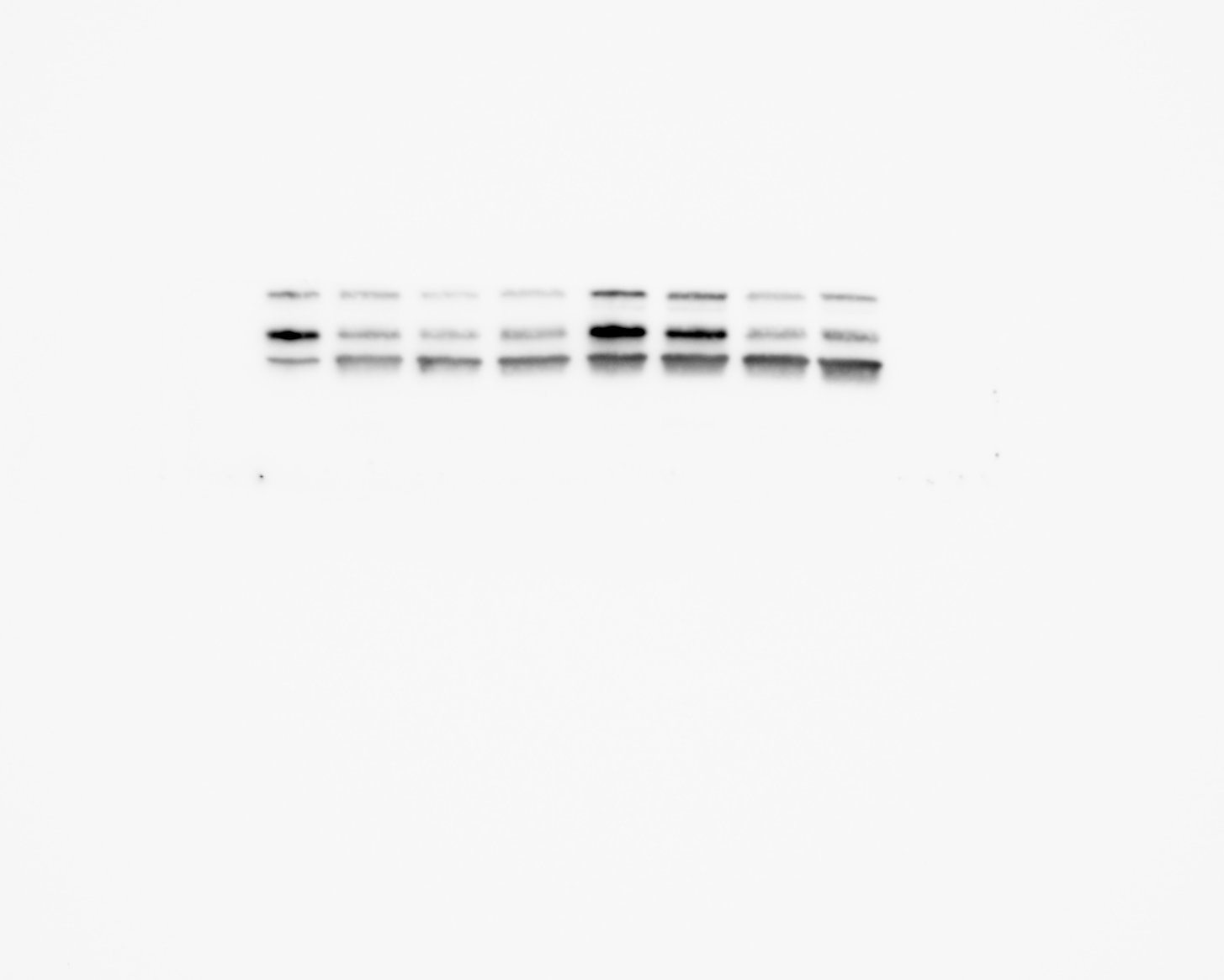

Supplement: Figure 4—source data 1. [file elife-90532-fig4-data1.zip › blots figure 4 raw/cdk1 (Chemiluminescence).tif]

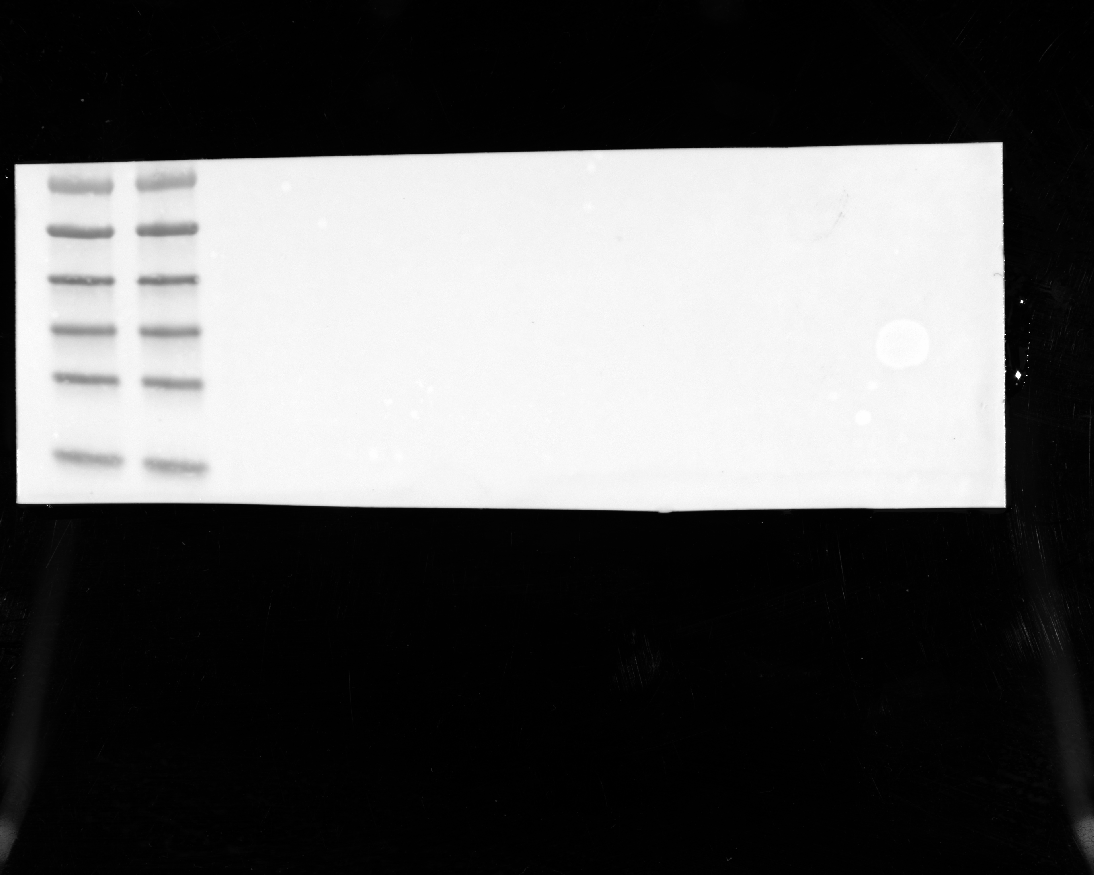

Supplement: Figure 4—source data 1. [file elife-90532-fig4-data1.zip › blots figure 4 raw/p jnk(Colorimetric).tif]

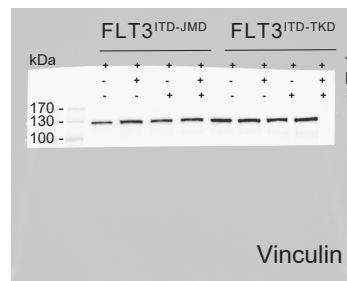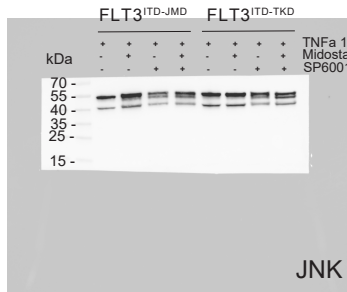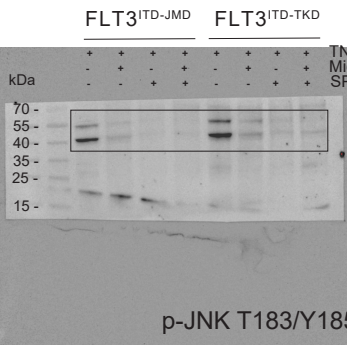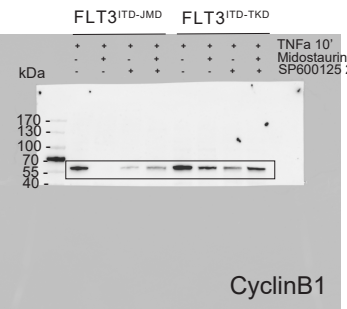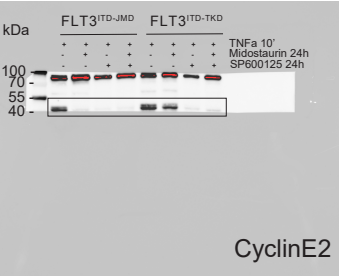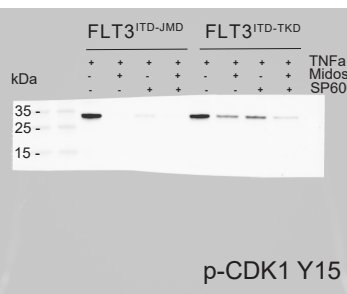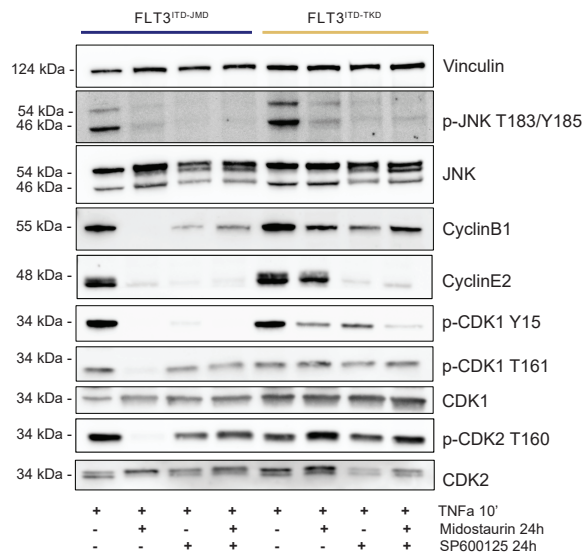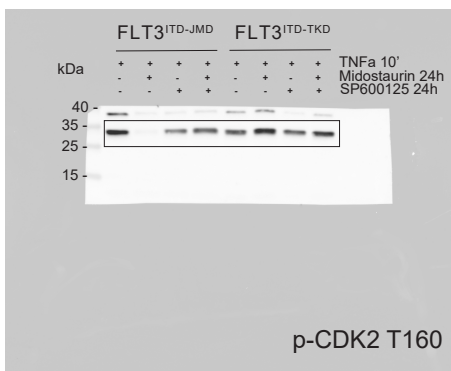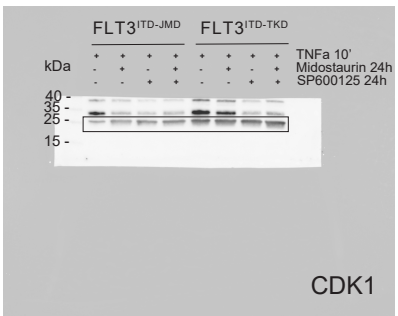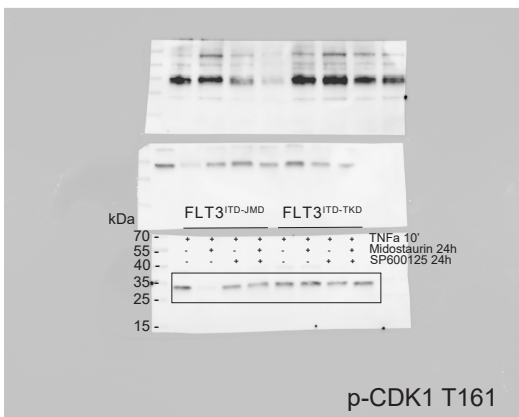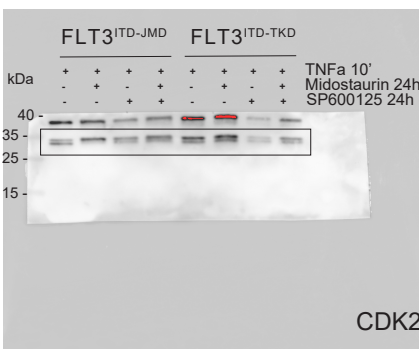

Figure 4

Supplement: Figure 4—source data 2. [file elife-90532-fig4-data2.pdf]
